# Protocol for using autoclaved intertidal sediment as a medium to enrich marine cable bacteria

**DOI:** 10.1016/j.xpro.2022.101604

**Published:** 2022-08-11

**Authors:** Cheng Li, Clare E. Reimers, Peter J. Chace

**Affiliations:** 1 College of Earth, Ocean, and Atmospheric Science, Oregon State University, Corvallis, OR 97333, USA

**Keywords:** Cell culture, Microbiology, Environmental sciences

## Abstract

Cable bacteria (CB) are non-isolated filamentous bacteria in the family of *Desulfobulbaceae*, known for fostering centimeter-long electron transfer in sediments with pronounced redox zonation. This protocol details steps to extract CB filaments from cultured natural sediment, inoculate autoclaved sediment with extracted filaments, and subsequently evaluate the growth and enrichment of CB. We also describe the approaches for collecting suitable sediment, preparing autoclaved sediment, and manufacturing glass needles and hooks for the extraction of CB.

## Highlights

• Prepare autoclaved sediment as an enrichment medium for cable bacteria
• Manufacture glass needles and hooks as tools to extract cable bacteria
• Video demonstration of cable bacteria extraction and autoclaved sediment inoculation
• Recover prolific cable bacteria biomass grown in autoclaved sediment

## Abstract (editor highlights)

Publisher’s note: Undertaking any experimental protocol requires adherence to local institutional guidelines for laboratory safety and ethics.

## Graphical abstract

**Figure:**
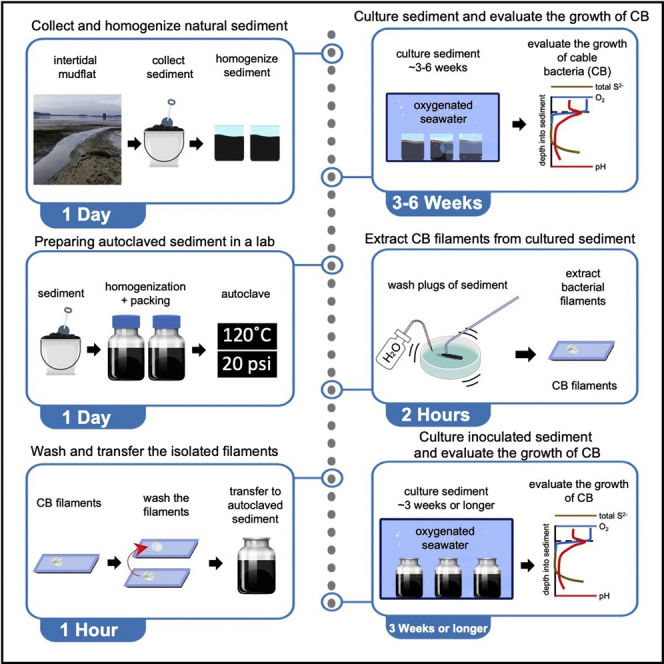

## Before you begin

Cable bacteria (CB) are as of yet non-isolated taxa within the family of *Desulfobulbaceae*, known for establishing centimeter-long filamentous networks in the uppermost layers of sediments with redox zonation (Pfeffer et al., 2012). The long distances spanned by individual filaments allow CB to access energy harvested from sulfide oxidation (½ H_2_S + 2H_2_O → ½ SO_4_^2-^ + 4e^-^ + 5H^+^) within sulfidic zones and oxygen reduction (O_2_ + 4e^-^ + 4H^+^ → 2H_2_O) within oxic zones (Kjeldsen et al., 2019; Pfeffer et al., 2012). To transfer electrons between the reactants of the two half-reactions and to acquire metabolic energy, CB construct electrically conductive fibers within their periplasmic envelope that extend longitudinally along filaments (Boschker et al., 2021; Cornelissen et al., 2018; Meysman et al., 2019). The periplasmic fibers and the associated structures passing charge onto and off the fibers are being investigated for a host of biomaterial applications, while the phylogeny and lifestyle behavior of CB are of fundamental interest in microbial ecology and environmental applications such as remediation of contaminants. In this protocol, we detail a simple approach utilizing autoclaved natural sediment as a medium to enrich and obtain CB biomass for easier study.

The first step we follow to enrich CB in autoclaved sediment is to cultivate CB in natural sediment from which CB filaments have been observed. The electrically connected but separated half-reactions thus create (or permit) a suboxic zone where no oxygen or sulfide is detected. The natural presence of CB and the geochemical signature of their associated suboxic zone has been observed in various surficial sediments from diverse habitats, including intertidal mudflats, mangroves, rivers, salt marshes, and certain bioturbated sediments exhibiting stable biogenic structures (Aller et al., 2019; Burdorf et al., 2016; Dam et al., 2021; Li et al., 2020a, 2020b; Malkin et al., 2014, 2017; Malkin and Meysman, 2015; Risgaard-Petersen et al., 2015; Trojan et al., 2016). Prior research has not only provided a list of locations where CB can be found (Table 1), but it suggests that the ideal sedimentary matrix to enrich CB should contain 1) dissolved hydrogen sulfide in pore waters below the sediment-water interface, 2) persistent dissolved oxygen in the overlying seawater, and 3) a stably stratified zonation of redox layers. Evidence from geographic DNA databases has continued to show the existence of CB in a multitude of settings. However, care must be taken when collecting from a local site. Estuaries are the most common access point for fresh culturing material and can exhibit a range of conditions. In addition to these being potentially dangerous places to collect samples, local conservation efforts may be in place to protect existing or restore historic, mudflats and adjacent habitats. Even if the volume of culturing mud collected is small, be sure to determine the impact of the collection. CB would appear to thrive in human-disturbed settings, and we recommend focusing on collections that are purely for biomaterial work in already disturbed, high sedimentation environments such as harbors. Bulk collection of material from conservation sites, seagrass meadows, and protected mudflats should be avoided.

**Table 1:** Locations listed in previous studies where cable bacteria can be found

Sediment type	Approximate salinity (PSU)	Location	Coordinates	Reference
**Marine or Brackish**				

Harbor sediments	20	Aarhus Harbor Denmark	56.14°N, 10.22°E	(Nielsen et al., 2010; Pfeffer et al., 2012)
Bay sediment	30	Aarhus Bay Denmark	56.10°N, 10.46°E	(Nielsen et al., 2010; Pfeffer et al., 2012)
Unvegetated intertidal sediment	28	Rattekaai Salt Marsh, the Netherlands	51.44°N, 4.17°E	(Malkin et al., 2014; Malkin and Meysman, 2015)
Muddy subtidal sediment	N/A	Station 130 in the Belgian Coastal Zone	51.27°N, 2.91°E	(Malkin et al., 2014)
Muddy sediment in a seasonally stratified coastal basin.	30	Marine Lake Grevelingen, the Netherlands	51.75°N, 3.89°E	(Malkin et al., 2014; Seitaj et al., 2015)
Muddy intertidal mudflat sediment	29	Sally’s bend, Yaquina Bay, Oregon, USA	44.63°N, 124.01°W	(Li et al., 2020a)
Subtidal deposits from downstream of a commercial oyster farm	29	Yaquina Bay, Oregon, USA	44.58°N, 123.99°W	(Li et al., 2020a)
Carbonate muddy bay sediment	25	Florida Bay, Florida, USA	25.12°N, 80.82°W	(Yin et al., 2021)
Muddy sediment within the bivalve reef	29	Northeast shore of the barrier island of Texel, Wadden Sea, The Netherlands	53.15°N, 4.90°E	(Malkin et al., 2017)
Muddy sediment bioturbated by the parchment worm	26–31	Great Peconic Bay, Long Island, New York, USA	40.95°N, 72.49°W	(Aller et al., 2019)
Muddy intertidal mudflat sediment bioturbated by mud shrimps	29	Idaho flat, Yaquina Bay, Oregon, USA	44.62°N, 124.04°W	(Li et al., 2020b)
Tidal sediment covered by a biofilm dominated by diatoms	28	Oosterschelde tidal inlet, the Netherlands	51.44°N, 4.17°E	(Malkin et al., 2014; Malkin and Meysman, 2015)
Seagrass sediment	30–32	The Swan River estuary, Australia	32.03°S, 115.76°E	(Martin et al., 2019)
Mangrove sediment	30–32	Western Port and Anderson Inlet, Australia	38.633°S, 145.183°E and 38.229°S, 145.309°E	(Burdorf et al., 2016)

**Freshwater or groundwater**				

Surface sediment of a lowland, hard-water stream	0	Giber Å, Eastern Jutland, Denmark	56.07°N, 10.17°E	(Risgaard-Petersen et al., 2015; Scholz et al., 2019)
Freshwater ponds sediment	0	Vennelystparken, Aarhus, Denmark	56.16°N, 10.21°E	(Kjeldsen et al., 2019)
Groundwater aquifer sediment contaminated by tar oil	0	Düsseldorf-Flingern, Germany	51.22°N, 6.82°E	(Müller et al., 2016, 2020)

### Harvest sediment that contains cable bacteria from an accessible location

**Timing: 1 day**

1. Identify a safely accessible location to harvest marine sediment away from protected habitats or highly polluted areas.
2. Collect the suboxic to the sulfidic zone of the sediment (usually between 0–30 cm depth) using a shovel or other digging tools.

***Note:*** The geochemical patterns created by the CB in porewater profiles can be measured onsite using microelectrodes before selecting a collection depth for the sediment. Details about how to perform onsite microelectrode profiling can be found in Malkin et al. (2014).

3. Put the sediment in a large container (e.g., a large bucket with a lid), gathering only as much as needed.
4. If possible, collect some overlying water to measure the salinity by using a conductivity-based salinity meter or a refractometer.
5. If enough overlying water is present, collect several liters or retrieve water from a nearby location with similar salinity in a different container.
6. Bring sediment and seawater to a laboratory.
7. Store the sediment and seawater at a refrigerated temperature (5°C or lower) or process the sediment right away as described in the next step.

**CRITICAL:** Prior to sampling, be sure to determine appropriate personal protective equipment for the collection location, such as boots and waders in cold environments. When sampling tidal locations, check tide tables prior to collection and be aware of your surroundings. Do not sample alone.

***Alternatives:*** Sediment can be collected by other retrieval tools such as push cores, sediment grabs, and traditional digging equipment.

### Homogenize sediment in a laboratory

**Timing: 1 day**

8. Sieve the stored sediment through a 0.5 mm mesh screen to remove any large fauna, shells, rocks, and debris, capturing sieved material into a clean receptacle.
9. Homogenize the sieved sediment by physical mixing.

***Note:*** Oxygen exposure can be reduced during the homogenization process by introducing a tube that flows nitrogen gas into the sediment.

10. Pack the homogenized sediment into containers with a large opening (e.g., a core liner or a wide-mouth glass bottle).

**CRITICAL:** The homogenization of sulfidic sediment may release hydrogen sulfide, a toxic chemical in its gaseous form. We highly recommend the use of personal protection equipment such as a sulfide-specific face respirator or conducting the homogenization process under a ventilation hood or in a very well-ventilated open space.

***Alternatives:*** A kitchen strainer with a similar mesh size can replace the use of a mesh screen. Nitrogen gas can be replaced by other inert gases such as helium and argon.

### Culture natural sediment and evaluate the growth of cable bacteria

**Timing: 3–6 weeks**

11. Place the containers into an aquarium filled with autoclaved seawater and keep the seawater aerated by using a commercially available aquarium air pump and bubbling stone.
12. Evaluate the growth and presence of CB by measuring the vertical chemical profiles of oxygen, sulfide, and pH.

***Note:*** The vertical chemical profiles of oxygen, sulfide, and pH that are characteristic of CB can be measured by microelectrodes. More details about performing microelectrode profiling in a lab can be found in Malkin et al. (2014) and Li et al., 2020a, Li et al., 2020b.

***Alternatives:*** Artificial seawater can also be used as the culture media in commercially available aquarium tanks. The presence of CB can also be verified by using microscopic techniques (e.g., scanning electronic microscopy and transmission electron microscopy) to identify their unique morphological features namely the longitudinal ridges and cartwheel cell-cell junctions (Li et al., 2020b; Pfeffer et al., 2012), molecular techniques (e.g., fluorescent in situ hybridization) (Li et al., 2020b; Malkin et al., 2014, 2017), and sequencing techniques (e.g., maker gene analysis and metagenomics) (Geelhoed et al., 2020; Kjeldsen et al., 2019; Li et al., 2020b; Trojan et al., 2016). The growth and density of CB can be evaluated using counts of stained filaments and quantitative polymerase chain reaction methods (Aller et al., 2019; Geelhoed et al., 2020).

### Prepare thin glass hooks and needles for extracting CB filaments

**Timing: 1 h**

13. Hold the two ends of a glass capillary tube and put the middle of the tube over the flame of a Bunsen burner.
14. Rotate the tube slowly to heat evenly.
15. When the middle of the glass tube becomes soft, quickly move the tube away from the flame and pull the two ends evenly to make a thin tapered mid-section. Troubleshooting.
16. Separate the glass tube into two pipettes by melting at the middle of the newly pulled section and shorten the thin end to a desired length.
17. Use heat from outside a lighter flame to bend the thin tip of each glass pipette needle into the shape of a hook.

**CRITICAL:** Wear gloves, do not touch hot glass, and be watchful to avoid burns (Methods video S1).

Methods video S1. Preparing glass needles and glass hooks

## Key resources table

**Table:** 

REAGENT or RESOURCE	SOURCE	IDENTIFIER
**Other**		

5 Gallon White Plastic Bucket & Lid	Amazon	https://www.amazon.com/dp/B075X4SPSG/ref=cm_sw_em_r_mt_dp_VRDRT98F03K0PSCWR5Q9
Heavy Duty Carbon Steel Garden Hand Shovel	Amazon	https://www.amazon.com/dp/B079P8HBY1/ref=cm_sw_em_r_mt_dp_TBCZYCT64JN2FYCHQM0N
Water and slip resistant Boots	VWR International LLC	Cat# 76104-862
Fisherbrand™ U.S. Standard Brass Sieves with Steel Cloth (0.5 mm)	Fisher Scientific LLC	Cat# 04-881-19
Stainless Steel Mesh Strainer	Amazon	https://www.amazon.com/dp/B007TUQF9O/ref=cm_sw_em_r_mt_dp_WWK4NAXVPA8JXS8166VS
Compressed nitrogen gas	Airgas	Part# NI 230LT22
Core tubes	McMaster-Carr	https://www.mcmaster.com/polycarbonate-tube/high-pressure-hard-plastic-tubing-for-air-and-water/
Core tube caps	Caplugs	https://www.caplugs.com/sleeve-caps-sc
Regular-Mouth Glass Mason Jars, 8-Ounce	Amazon	https://www.amazon.com/dp/B096WBXF9R/ref=cm_sw_em_r_mt_dp_A98C3JFEEYEEV7NC1CVK
10-gallon aquarium	Aqueon	https://www.aqueon.com/products/aquariums/standard-glass-rectangle-aquariums
AQUANEAT Aquarium Air Pump 300GPH	Amazon	https://www.amazon.com/dp/B088THVBZF/ref=cm_sw_em_r_mt_dp_X5BV4775MT61X2WPPGXH?_encoding=UTF8&psc=1
Standard Bunsen Burners	VWR International LLC	Cat# 470148-926
Capillary Melting Point Tubes, 0.20 mm Wall Thickness	VWR International LLC	Cat# 80061-548
Media/Storage Bottles with GL Screw Caps, 1 L, With Cap	VWR International LLC	Cat# 10754-820
Media/Storage Bottles with GL Screw Caps, 100 mL, With Cap	VWR International LLC	Cat# 10754-814
Syringe 1 cc	VWR International LLC	Cat# 100500-888
VWR® Petri Dishes, Glass	VWR International LLC	Cat# 75845-544
VWR VistaVision™ Microscope Slides, Plain	VWR International LLC	Cat# 16004-422
Fisherbrand™ Nichrome Inoculating Needles with Handles	Fisher Scientific LLC	Cat# 14-956-101

## Step-by-step method details

### Prepare autoclaved sediment and seawater in a laboratory

**Timing: 1 day**

The following procedure describes the steps required to prepare autoclaved sediment from aliquots of the sediment previously harvested.

1. As was done for incubations, sieve the stored sediment through a 0.5 mm mesh screen to remove large fauna, rocks, and debris.
2. Homogenize the sieved sediment by physical mixing. Troubleshooting.

***Note:*** Oxygen exposure can be reduced during the homogenization process by introducing a tube that flows nitrogen gas into the sediment.

3. Fill 100 mL autoclave-safe glass bottles with homogenized sediment to about ¾ of the volume.
4. Fill 1000 mL autoclave-safe bottles with seawater to about ¾ of the volume.

***Note:*** Sterilized artificial seawater can be used as well.

5. Autoclave the glass bottles at 120°C for 1–2 h.
6. Allow the autoclaved sediment and seawater to cool down to about 25°C before use.

***Note:*** Autoclaved sediment can also be stored and sealed at about 25°C until inoculation. The containers remain sealed and are stored in a cool place, for up to two weeks in this study.

**CRITICAL:** The homogenization of sulfidic sediment and mishandling of iron sulfide may release hydrogen sulfide, a highly toxic chemical in its gaseous form. We again recommend the use of personal protective equipment such as a sulfide-specific face respirator when in enclosed spaces, and the homogenization process should be conducted under a ventilation hood or in an open well-ventilated space. The autoclaved sediment and seawater should be prepared at least one day to two weeks ahead of the following steps.

***Alternatives:*** Nitrogen gas can be replaced by other inert gases such as helium and argon.

### Extract cable bacteria filaments from the cultured natural sediment

**Timing: 2 h**

The following procedure describes the steps required to isolate CB filaments from the cultured natural sediment matrix.

**Figure 1:**
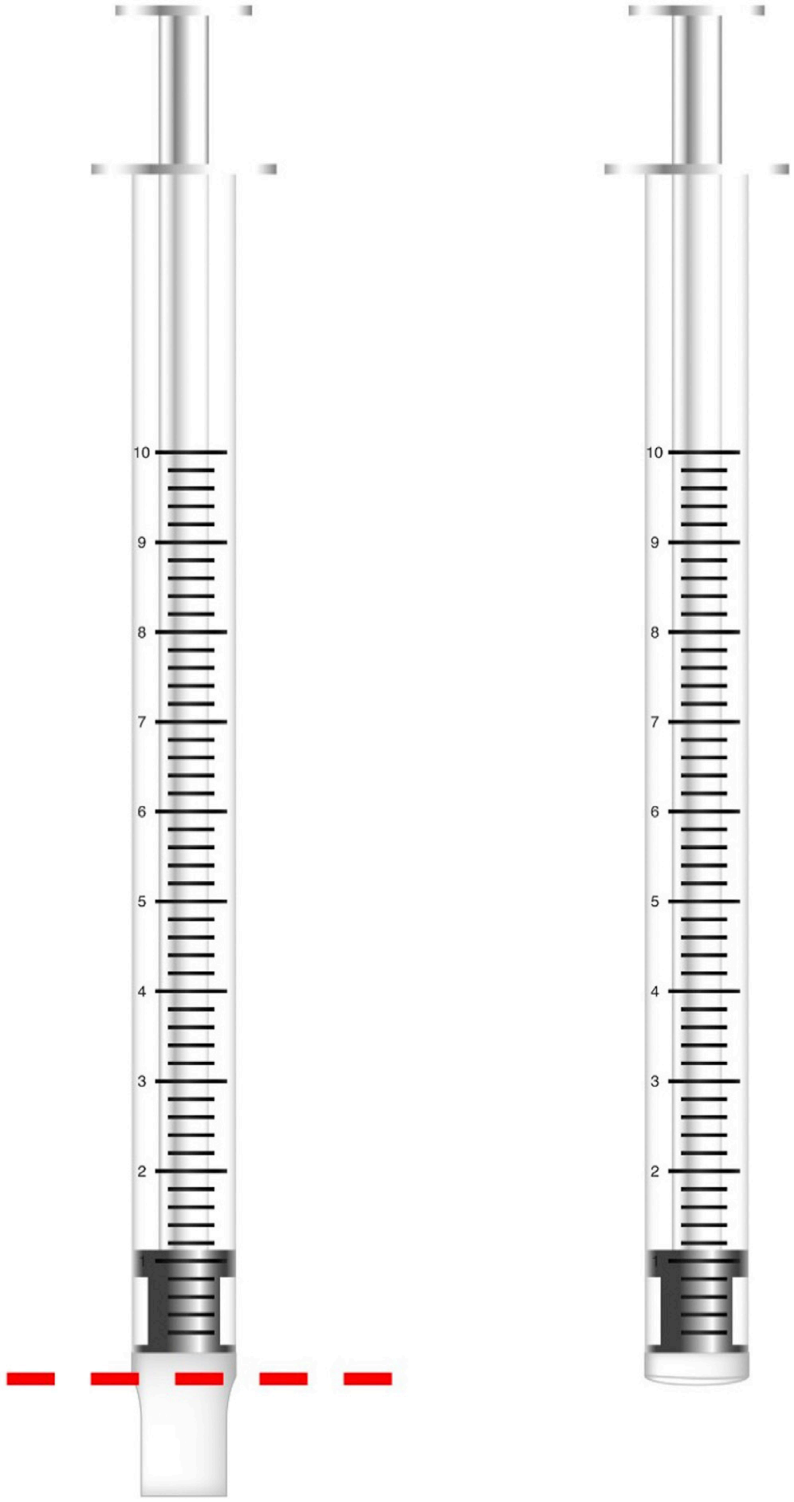
Graphical demonstration of the preparation of a cut-off syringe Left: cutting the tip of the 1cc syringe to allow the proper intake of a sediment plug (the red line indicates where to make the cut); right: the finished look of the cut-off syringe.
8. Reduce the sediment volume around the sampled mass of CB filaments by gentle washing Troubleshooting.
  a. Place the extracted plugs of sediment in a glass Petri dish.
  b. Add seawater or artificial seawater to the Petri dish.
  c. Gently shake the Petri dish to allow the sediment particles to separate from the CB filaments.
  d. Remove sediment-laden water from the Petri dish.
  e. Repeat b–d until the filamentous structure of CB can be observed, and the amount of adhering sediment grains can no longer be reduced (Methods video S2).

***Note:*** Deionized water can be used in this step as well.

7. Take sub-cores (sediment plugs approximately 0.5 cm in diameter and 1 cm long) from the cultured sediment by using cut-off 1 mL syringes (Figure 1).

9. Use the fabricated glass hook to pick CB filaments from the remainder of the washed sediment plug(s). Troubleshooting.
  a. Push the hook gently into a suboxic zone of the remaining washed sample, which can be identified by its grayish color.
  b. Swirl the hook gently several times (3–7).
  c. Slowly pull the hook away from the sample and look for filamentous structures of CB connecting (extending between) the hook and the remainder of the sediment.
  d. Collect the filaments by pulling the hook apart from the sediment (Methods video S2).

**Figure 2:**
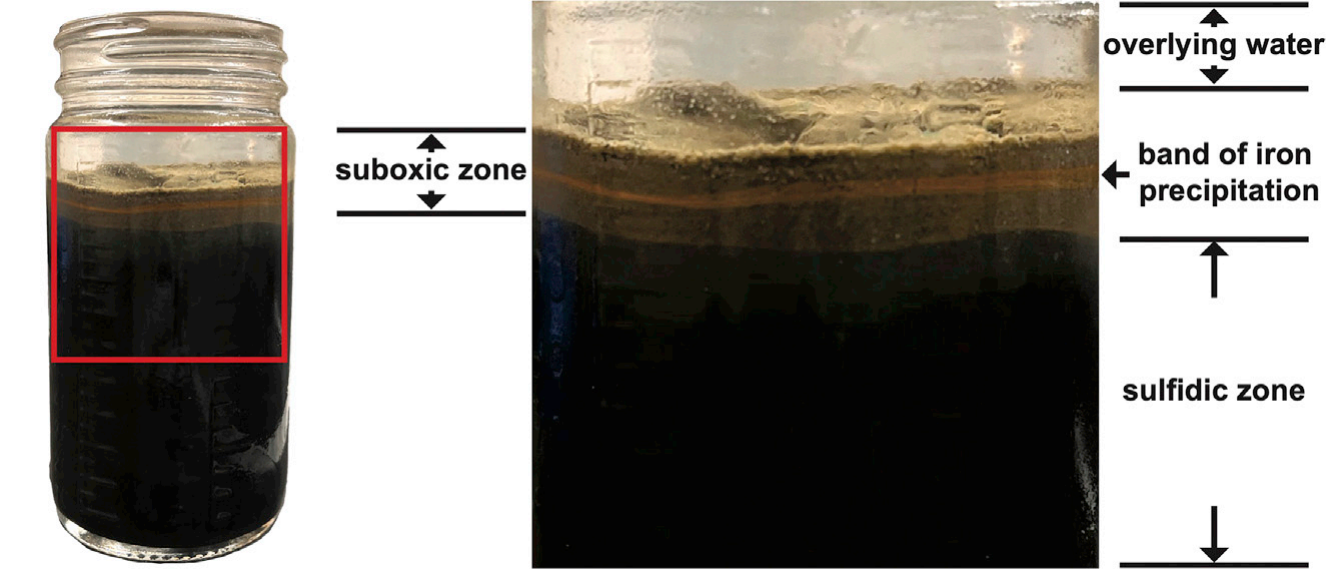
The stratified zonation of topmost sediment inhabited by cable bacteria

***Note:*** After being pulled out from the sediment, a mass of CB will shrink onto the hook and appear like a “glob”.

**CRITICAL:** Samples should be taken from cultured sediments which have developed a pronounced suboxic zone evident by color changes from black to gray and the presence of a band of iron precipitation at the subsurface (Figure 2).

Methods video S2. Extracting cable bacteria filaments from the natural sediments

### Wash and transfer the isolated filaments into the autoclaved sediment

**Timing: 1 h**

The following procedure describes the steps followed to further wash the isolated CB filaments and transfer the filaments into the autoclaved sediment.

10. Transfer the isolated CB filaments to a droplet of the washing solution placed on a microscopic slide (Methods video S3).
  a. Use a clean glass needle to scrape the biomass off the glass hook.
  b. Resuspend the biomass in the droplet of the washing solution.

***Note:*** Washing solution can be autoclaved seawater, artificial seawater, phosphate-buffered saline (PBS) solution, or DI water.

11. If necessary, repeat step 10 until the biomass appears free of sediment particles.
12. Use another clean glass needle to pick up the washed biomass and transfer the biomass into the surface layer of the autoclaved sediment (Methods video S3).
13. Increase the probability of transferred CB survival by performing multiple transfers into the surface layer of the autoclaved sediment.

***Optional:*** A sterilized inoculation needle can be used to transfer the CB biomass.

Methods video S3. Washing and transferring cable bacteria filaments to autoclaved sediment

### Culture inoculated sediment and evaluate the growth of cable bacteria

**Timing: 3 weeks or longer**

The following procedure describes the steps required to culture inoculated sediment and to evaluate the growth of cable bacteria.

14. Slowly submerge the inoculated sediment into an aerated aquarium filled with an autoclaved medium.
15. Culture the inoculated sediment. Troubleshooting.
16. Evaluate the growth and presence of CB by measuring the vertical chemical profiles of oxygen, sulfide, and pH.

***Note:*** The vertical chemical profiles of oxygen, sulfide, and pH that are characteristic of CB can be measured by the microelectrodes. More details about performing microelectrode profiling in a lab can be found in Malkin et al. (2014) and Li et al., 2020a, Li et al., 2020b.

***Alternatives:*** Artificial seawater can also be used as the culture media in commercially available aquarium tanks. The presence of CB can also be verified by using microscopic techniques (e.g., scanning electronic microscopy and transmission electron microscopy) to identify their unique morphological features namely the longitudinal ridges and cartwheel cell-cell junctions (Li et al., 2020b; Pfeffer et al., 2012), molecular techniques (e.g., fluorescent in situ hybridization) (Li et al., 2020b; Malkin et al., 2014, 2017), and sequencing techniques (e.g., maker gene analysis and metagenomics) (Geelhoed et al., 2020; Kjeldsen et al., 2019; Li et al., 2020b; Trojan et al., 2016). The growth and density of CB can be evaluated using counts of stained filaments and quantitative polymerase chain reaction methods (Aller et al., 2019; Geelhoed et al., 2020).

## Expected outcomes

**Figure 3:**
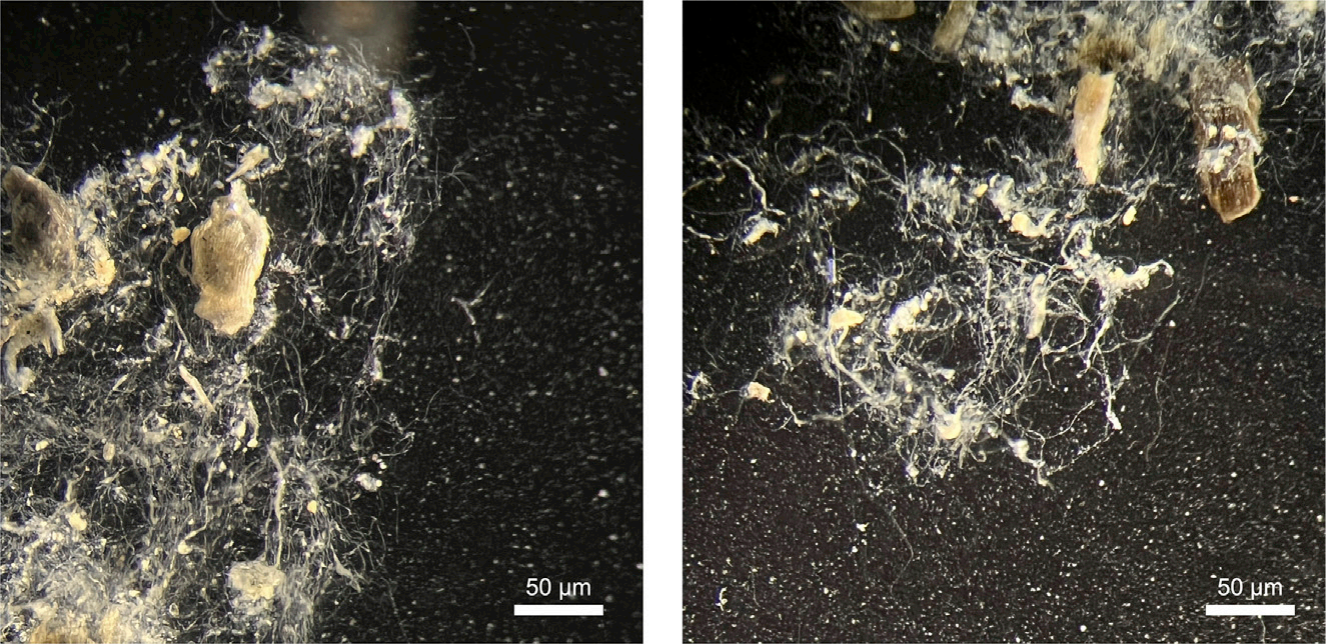
Light microscope images of the cable bacteria filaments enriched in autoclaved intertidal sediments originally collected from Yaquina Bay, OR

**Figure 4:**
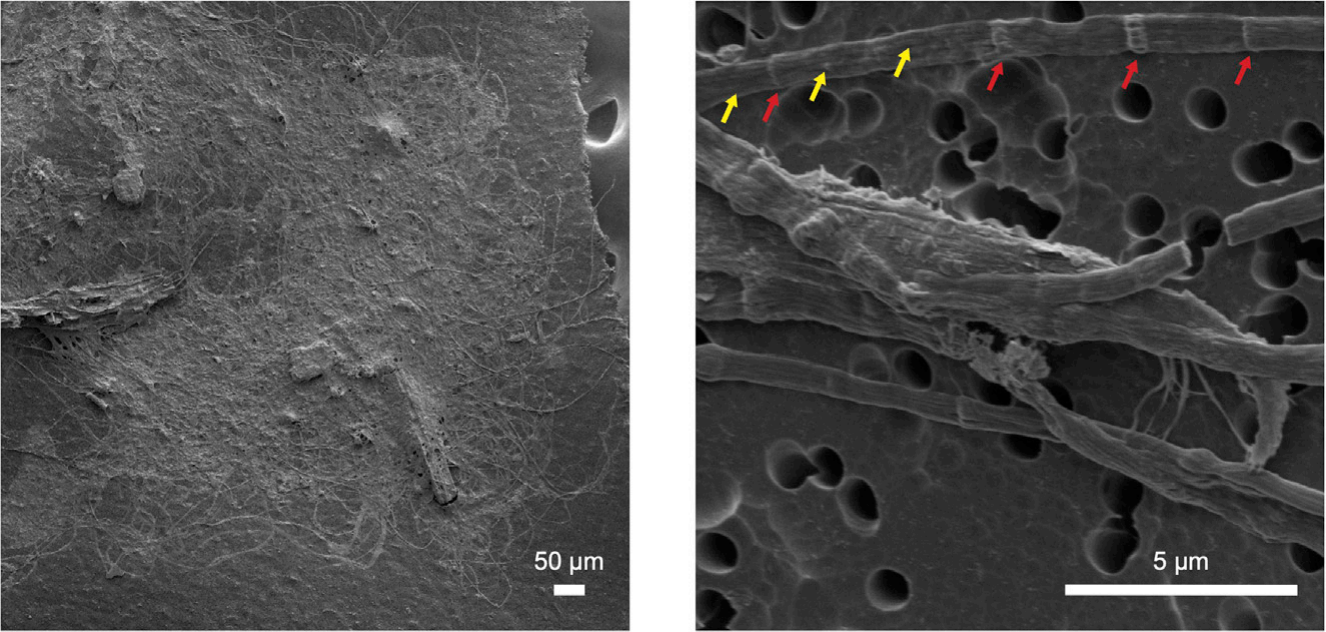
Images of the enriched cable bacteria filaments captured by a scanning electron microscope (Helios 650 Ultra Resolution Dual Beam) Left: a bundle of the extracted filaments; right: tangled cable bacteria filaments within the bundle exhibiting the typical morphological features (indicated on the topmost filament, yellow arrows: the longitudinal ridges, and red arrows: the cartwheel-like cell-cell junctions).

Electrogenic sulfur oxidation catalyzed by CB will significantly alter porewater chemistry, and these changes can be utilized as indicators of successful inoculation (Malkin et al., 2014; Nielsen and Risgaard-Petersen, 2015; Pfeffer et al., 2012). The signature of spatially separated and yet electrically connected sulfide oxidation/oxygen reduction is a pH minimum at depth and a pH maximum near the surface of the sediment column. The development of the two pH extremes and a suboxic zone can be measured by chemical profiling using microelectrodes (see Unisense microelectrode products and studies mentioned above) as early as 2 weeks after inoculation. Secondly, at the pH minimum, buried minerals such as iron are being redissolved and will diffuse towards the sediment-water interface (Otte et al., 2018b; Rao et al., 2016; Seitaj et al., 2015; Sulu-Gambari et al., 2016a, 2016b). At the pH maximum, iron is subsequently oxidized and reprecipitated in the presence of oxygen. Over time, starting from weeks 4–5, the precipitated minerals can be visualized as a distinctive band of orange color at the subsurface (Figure 2), suggesting that the enrichment culture is mature. Finally, established microscopic methods mentioned in the previous sections can be applied to examine the filamentous biomass of CB (Figures 3 and 4). The longitudinal ridges and cartwheel cell-cell junctions of the filaments can be identified by scanning electron microscopy (Figure 4). The purity and the phylogeny of the cultured CB can be assessed by molecular and sequencing techniques (e.g., marker gene analysis and metagenomics) (Geelhoed et al., 2020; Kjeldsen et al., 2019; Trojan et al., 2016).

## Limitations

Natural sediment is a complex matrix that contains various microorganisms, some of which may evade the sterilization of an autoclave, or leave their DNA behind (Otte et al., 2018a). The biomass of CB used as inoculum is almost certain to contain other microorganisms. The method in the present study is therefore designed to ensure the enrichment of CB and its dominant growth in culture, not to achieve true isolation of CB.

## Troubleshooting

### Problem 1

Unable to make a thin tapered mid-section when pulling the heated glass tube. (Related to step 15 in “prepare thin glass hooks and needles for extracting CB filaments”).

### Potential solution

Different types of glass tubes may have different softening temperatures. However, it is critical to quickly move the tube away from the flame and pull when the middle of the glass tube becomes soft.

### Problem 2

Low to no sulfide in sediment after autoclave. (Related to step 2).

### Potential solution

The continuously boiling during autoclaving will likely diminish the concentration of total sulfides in the sediment. To ensure sustainable growth of CB, iron sulfide serving as an additional sulfide source can be added to the sediment (1:100 weight to weight) during homogenization. Alternatively, other sulfide sources such as sodium sulfide can be supplied to the sediment after the autoclave process.

### Problem 3

Unable to extract filaments from the reduced volume sub-core from natural cultured sediments. (Related to steps 7 and 8).

### Potential solution

There are various possible reasons for this problem. Firstly, the growth of CB in the topmost layer of cultured sediment is often heterogeneous. Therefore, some spots may contain little to no CB biomass. Taking another sub-core in a different location can be a potential solution. Secondly, when in less abundance, CB filaments are easily broken and overlooked. Use gentle force for washing and a thin glass hook with a long neck for extracting.

### Problem 4

A suboxic zone does not appear to develop during the first few weeks of culture in autoclaved sediment. (Related to step 15).

### Potential solution

Keep inoculating the sediment with freshly extracted CB biomass to enhance the survival and growth of CB. Additionally, burying the CB biomass in the topmost layer of the autoclaved sediment (∼1–2 mm depth) may also enhance the survival rate.

### Problem 5

A limited amount of CB biomass is found in the inoculated autoclaved sediment. (Related to step 15).

### Potential solution

Possible reasons are that the conditions required for CB biomass to grow have not been met. When preparing the autoclaved sediment and inoculum, ensure that 1) the sediment contains sulfide minerals as a substrate for the CB and 2) the filaments extracted from the cultured natural sediment are CB. If the sediments do not contain enough sulfide, iron sulfide can be supplied to the sediment before autoclave. The identity of the inoculum can be verified by using microscopic or phylogenetic analyses. Additionally, ensure the oxygen concentration is sufficient in the overlying water.

## Resource availability

### Lead contact

Further information and requests for resources and reagents should be directed to and will be fulfilled by the lead contact, Cheng Li (Cheng.Li@oregonstate.edu).

### Materials availability

This study did not generate new unique reagents.

## Data Availability

This study did not generate datasets/code.
